# Evaluation of Toxicity and Oxidative Stress of 2-Acetylpyridine-*N*(4)-orthochlorophenyl Thiosemicarbazone

**DOI:** 10.1155/2022/4101095

**Published:** 2022-03-19

**Authors:** Andressa Brito Lira, Gabrieli Lessa Parrilha, Gabriela Tafaela Dias, Fernanda Samara de Sousa Saraiva, Gabriel Corrêa Veríssimo, Rayane Siqueira de Sousa, Teresinha Gonçalves da Silva, Abrahão Alves de Oliveira Filho, Adriano Francisco Alves, Elaine Maria de Souza-Fagundes, Heloisa Beraldo, Maria Aparecida Gomes, Margareth de Fatima Formiga Melo Diniz

**Affiliations:** ^1^Programa de Pós-Graduação em Produtos Naturais e Bioativos Sintéticos, Universidade Federal da Paraíba, João Pessoa, PB, Brazil; ^2^Departamento de Química, Instituto de Ciências Exatas, Universidade Federal de Minas Gerais, Belo Horizonte, MG, Brazil; ^3^Departamento de Fisiologia e Biofísica, Universidade Federal de Minas Gerais, Belo Horizonte, MG, Brazil; ^4^Departamento de Ciências Farmacêuticas, Universidade Federal de Minas Gerais, Belo Horizonte, MG, Brazil; ^5^Departamento de Antibióticos, Universidade Federal de Pernambuco, Recife, PE, Brazil; ^6^Unidade Acadêmica de Ciências Biológicas, Centro de Saúde e Tecnologia Rural, Universidade Federal de Campina Grande, Patos, PB, Brazil; ^7^Departamento de Fisiologia e Patologia, Universidade Federal da Paraíba, João Pessoa, PB, Brazil; ^8^Departamento de Parasitologia, Instituto de Ciências Biológicas, Universidade Federal de Minas Gerais, Belo Horizonte, MG, Brazil; ^9^Departamento de Ciências Farmacêuticas, Universidade Federal da Paraíba, João Pessoa, PB, Brazil

## Abstract

Thiosemicarbazones are well known for their broad spectrum of action, including antitumoral and antiparasitic activities. Thiosemicarbazones work as chelating binders, reacting with metal ions. The objective of this work was to investigate the *in silico*, *in vitro*, and *in vivo* toxicity and oxidative stress of 2-acetylpyridine-*N*(4)-orthochlorophenyl thiosemicarbazone (TSC01). The *in silico* prediction showed good absorption by biological membranes and no theoretical toxicity. Also, the compound did not show cytotoxicity against Hep-G2 and HT-29 cells. In the acute nonclinical toxicological test, the animals treated with TSC01 showed behavioral changes of stimulus of the central nervous system (CNS) at 300 mg/kg. One hour after administration, a dose of 2000 mg/kg caused depressive signs. All changes disappeared after 24 h, with no deaths, which suggest an estimated LD50 of 5000 mg/kg and GSH 5. The group treated with 2000 mg/kg had an increase of water consumption and weight gain in the second week. The biochemical parameters presented no toxicity relevance, and the analysis of oxidative stress in the liver found an increase of lipid peroxidation and nitric oxide. However, histopathological analysis showed organ integrity was maintained without any changes. In conclusion, the results show the low toxicological potential of thiosemicarbazone derivative, indicating future safe use.

## 1. Introduction

Thiosemicarbazones have versatile biological properties, the ability of coordination of transition metal ions, and general formula as R1R2CH = N‐NH‐(C = S)‐NH2 [1]. Thiosemicarbazones belong to a class of substances well known for their important applications in the research of new drug candidates, due to their broad spectrum of action [2, 3]. Some of the biological applications include antitumoral [3, 4], antiparasitic [5, 6], antifungal [7], antivirals [8], and antibacterial [9, 10] activity.

Usually, thiosemicarbazones work as chelating binders, reacting with metal ions by bonding through thiocarbonyl sulfur and azomethine nitrogen atoms [1]. The thiosemicarbazone mechanism is unknown. However, some of the hypotheses are generation of reactive oxygen species (ROS) through the formation of toxic complexes when bound to the cellular metals [11] and/or impeding the uptake of iron which inhibits various enzymes such as ribonucleotide reductase. Iron is essential in the cancer cell proliferation and generation of ROS [12].

The overproduction of ROS leads to oxidative damage, which is toxic to many biological functions, as they react with proteins, DNA, and lipids, causing cell and tissue injury [13, 14]. It is well known that oxidative stress is closely related to the inflammation process. Inflammation is a natural protective reaction to stimuli such as injury, infection, trauma, toxins, and other imbalances. However, uncontrolled inflammation promotes cell apoptosis and a large number of diseases [15, 16]. As all chemical compounds have some toxicity, knowing the side effects and interactions is vital [15].

It was recently proven that some TSC derivatives have a protective effect on cadmium toxicity in the liver and kidney of rats. The main mechanisms of action may be the antioxidant activity and chelating binder [17]. During the drug development process, drug toxicity evaluation is essential and required by international health authorities' agencies [18]. It takes approximately US$2.6 billion to release a new drug to market, and nonclinical and clinical toxicity tests are more than one-third of the total drug development cost [18, 19]. Pathological adverse effects during nonclinical steps cause difficulty in advancement to clinical trials, because of the translatability to humans [18]. Most failures during clinical trials are related to safety reasons [19].

**Figure 1:**
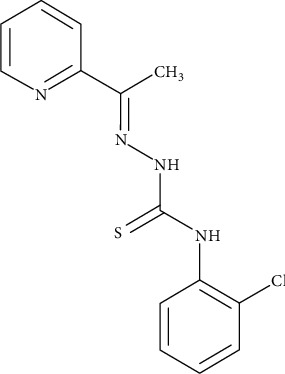
Structural representation of 2-acetylpyridine-*N*(4)-orthochlorophenyl thiosemicarbazone (TSC01).

Taking into account the need for new safe bioactive molecules against parasites, the objective of this study was to determine *in silico*, *in vitro*, and *in vivo* toxicity of 2-acetylpyridine-*N*(4)-orthochlorophenyl thiosemicarbazone (TSC01) (Figure 1) following international guidelines. TSC01 is a thiosemicarbazone derivative that presents antifungal activity against Candida spp. [7] and antimalarial [20] and antitrypanosomal activities [21]. TSC01 has notably demonstrated antitumor activity against breast and glioma cells [22, 23] which suggests possible use for brain tumor treatment.

## 2. Materials and Methods

### 2.1. General Procedure for the Synthesis of 2-Acetylpyridine-*N*(4)-orthochlorophenyl Thiosemicarbazone (TSC01)

2-Acetylpyridine-*N*(4)-orthochlorophenyl thiosemicarbazone was prepared as previously described [20].

### 2.2. Human Cell Line

The cell lines used were Hep-G2 and HT-29 cells. These cell lines were used to evaluate the chemical cytotoxicity [24]. The cells Hep-G2 and HT-29 were provided by Prof. Marcel Leist (University of Konstanz/Germany). Cells were cultured in DMEM, containing 10% fetal bovine serum (Gibco BRL, Grand Island, NY), supplemented with 100 U/mL penicillin and 100 *μ*g/mL streptomycin (GIBCO BRL, Grand Island, NY). All cultures were maintained at 37°C in a humidified incubator with 5% CO_2_. Cells were split twice a week.

### 2.3. Animals

*In vivo* assays were performed using females, *Mus musculus* mice, of Balb/c strain of the Fiocruz Pernambuco Vivarium, weighing between 22 and 29 g. The mice were kept at a temperature of 21 ± 1°C, with a light and dark cycle of 12 h, and fed with pellet-type feed and water ad libitum. The Committee on Ethics in Animal Use of UFPB approved the experimental protocol with registration no. 099/2016.

### 2.4. *In Silico* Study: Molinspiration

Molecular properties were calculated, based on molecular descriptors using Lipinski's rule of five, using the Molinspiration Online Property Calculation Toolkit software (http://www.molinspiration.com/). The molecular properties were represented by the partition coefficient, log *P*, hydrophilia, and number of hydrogen bonding donors and acceptors [25].

### 2.5. *In Silico* Study: Prediction of Pharmacokinetic and Toxicological Parameters

The pharmacokinetic parameters and theoretical toxicological ADMET (absorption, distribution, metabolism, excretion, and toxicity) were calculated using the admetSAR tool. The parameters were blood-brain barrier permeability, Caco-2 permeability, absorption in the intestine, substrates and inhibitors of cytochrome enzymes, and inhibitors of renal cation transport. Through this tool, metabolism using certain cytochrome P450 enzymes was evaluated comparing whether the compounds were substrates for cytochromes CYP450 2D6, CYP450 3A4, and CYP450 2C9; whether they were inhibitors of cytochrome CYP450 1A2, CYP450 2C9, CYP450 2D6, CYP450 2C19, and CYP450 3A4; and whether there was cytochrome inhibition promiscuity [26].

### 2.6. In Vitro: Cytotoxicity in Mammalian Cell Lines

Measurements of the cytotoxic effect of thiosemicarbazones on viability of Hep-G2 and HT-29 were performed using resazurin assay as previously described [27]. Resazurin is a weak fluorescent dye that is reduced by viable cells to resorufin, a strong fluorescent compound. Its fluorescence intensity is proportional to the number of viable cells in a culture. In short, cells were seeded at 1 × 10^4^ cells/well, in 96-well plates, and preincubated in a 95% air-humidified atmosphere with 5% CO_2_ for 24 h at 37°C to allow for cell stabilization. TSC01 was evaluated over a twofold serial dilution concentration (100-0.78 *μ*M), following incubation for an additional 24 h. DMSO at 0.5% was used as negative control (solvent control). After incubation, 20 *μ*L of 50 *μ*g mL^−1^ resazurin (Sigma-Aldrich, USA) solution was added per well after the cells were incubated for 48 h. The plates were incubated at 37°C under 5% CO_2_ and 100% humidity for 3 h. Fluorescence was read in a plate reader (VarioScan, Thermo Scientific®), at two wavelengths: excitation at 530 nm and emission at 590 nm. The number of viable cells correlated with the percentage of resazurin reduction and is expressed as percent cell viability as follows: %cell viability : (fluorescence of the assayed sample − fluorescence of the blank sample) × 100%/(control cell fluorescence). No interaction was observed among medium, compounds, and resazurin.

### 2.7. In Vivo: Acute Oral Toxicity Test

The toxicological test was performed according to OECD Guideline 423 with modifications [28]. The test started with a dose of 300 mg/kg, and subsequent repetition was executed if there were one or less death events. If the second group administered the 300 mg/kg again resulted in one or no deaths, the dose was increased to 2000 mg/kg, ending with another repetition when there were one or no deaths. Six Balb/c females per group were utilized, and TSC01 was administered by gavage in a single dose on day 1 in different doses per group. The control group was administered the dilution vehicle orally. After oral administration, behavioral observation was made at 30, 60, 90, 120, 180, and 240 minutes for pharmacological effect signs on the central nervous system. Daily food and water consumption were monitored, and body weight was documented on day 0 (before dosing), day 7, and day 14. After 14 days, euthanasia of the animals was conducted by excess anesthetic administration (20 mg/kg xylazine and 500 mg/kg ketamine). Blood samples were withdrawn for biochemical parameter analysis. Organ (heart, lung, stomach, spleen, liver, and kidneys) weight was measured, followed by histopathological examinations.

#### 2.7.1. Biochemical Parameters

Serum levels of urea, creatinine, aspartate aminotransferase (AST), and alanine aminotransferase (ALT) were determined.

#### 2.7.2. Anatomopathological and Histopathological Evaluation

After euthanasia, the organs were analyzed macroscopically; resections were executed with a sequential weighing of the heart, lung, stomach, liver, spleen, and kidneys (severed by sagittal incision). The organ indices were calculated pursuant to the formula index = weight of component (mg)/animal′s weight (g). The tissue sections were fixed in a formaldehyde solution of 10% and after 72 hours were prepared for histopathological processing: dehydration in an increasing alcohol series (70% to 100%), diaphanization in xylol, and impregnation with inclusion in paraffin. The organs were sectioned with a thickness of 3.0 *μ*m, mounted on slides, stained with hematoxylin and eosin, and then examined under an optical microscope at 200 and 400x.

#### 2.7.3. Determination of NO Levels

The concentration of nitrite in the homogenate (100 mg/mL in 150 mM phosphate buffer, pH 7.4) was used as an index of nitric oxide production through the Griess reaction, where 50 *μ*L of sample was incubated for 10 min with 50 *μ*L of Griess solution, protected from light. Absorbance was measured at a wavelength of 560 nm using a microplate reader, and the nitrite concentration was determined by comparing the absorbance of the sample to a standard curve for sodium nitrite [29].

#### 2.7.4. Determination of MDA Levels

The degree of lipid peroxidation was estimated by determining the levels of malondialdehyde (MDA), through the thiobarbituric acid reactive substances (TBARS) test, using the method described by [30]. Tissues were homogenized (100 mg/mL) in 150 *μ*M phosphate buffer (pH 7.4). After homogenization, the samples were mixed with 1 mL of 10% trichloroacetic acid and centrifuged at 10,000 rpm/15 min/4°C. Then, the supernatant was incubated with 500 *μ*L of 1.2% thiobarbituric acid. The mixture was brought to the boiling water bath (95°C/30 min). After the samples had cooled down, they were placed in a 96-well plate and read using a microplate reader (535 nm). Results were expressed in nmol MDA/mg tissue.

#### 2.7.5. Determination of GSH Levels

The determination of GSH concentration is based on the reaction of Ellman's reagent, 5,5′-dithiobis (2-nitrobenzoic acid) (DTNB), with the free thiol, resulting in a mixed disulfide plus 2-nitro-5 acid-thiobenzoic, and measurement of the reaction product formed is made by spectrophotometric reading. For GSH quantification, an aliquot of tissue homogenate, prepared in 0.02 M EDTA (1 mL/100 mg tissue), was mixed with 50 *μ*L distilled water+10 *μ*L 50% trichloroacetic acid and centrifuged at 5000 rpm/15 min/4°C. Then, 60 *μ*L of the supernatant was collected and 25 *μ*L of 0.4 M Tris buffer plus 20 *μ*L of 0.01 M DTNB was added, 1 minute after the reaction; a color reading was performed in a microplate reader at 412 nm [31].

#### 2.7.6. Determination of MPO Levels

Tissues were homogenized (1 mL/100 mg) in a 0.5% hexadecyltrimethylammonium bromide (HTAB) solution. Myeloperoxidase activity was evaluated according to the protocol described by [32], by reacting the samples with 200 *μ*L of reaction solution (o-dianisidine hydrochloride -0.167 mg/mL, 50 mM sodium phosphate buffer, and 0.0005% hydrogen peroxide). After incubation, the reaction was read in a microplate reader at a wavelength of 450 nm.

### 2.8. Statistical Analysis

Values are expressed as mean ± standard deviation (SD). The cytotoxicity, the results normalized by vehicle (DMSO at 0.5%), and half-maximal inhibitory concentration (IC_50_) values were obtained from concentration–effect curves. Three experiments were performed in triplicate. In the in vivo and ex vivo procedures, the statistical significance between groups was determined using a one-way analysis of variance (ANOVA), followed by Dunnett's test, with *P* < 0.05 indicating significance. All statistical analyses were performed using GraphPad Prism 6.0 and 7.0 (GraphPad Software, Inc., La Jolla, CA, USA).

## 3. Results and Discussion

### 3.1. In Silico: Bioinformatics

The process of drug discovery and development is lengthy and resource-intensive, resulting in high cost and risk. *In silico* models have reduced costs and timelines, because they facilitate the elimination of compounds with potential adverse effects or poor pharmacokinetics [33]. Lipinski parameters assist to establish if compounds can be properly absorbed and penetrate biological systems and therefore have a good oral bioavailability [34]. The desirable drug candidates must comply with at least four of the five parameters such as (a) number of hydrogen bond acceptor groups (nALH) ≤ 10, (b) number of hydrogen bond donor groups (nDLH) ≤ 5, (c) molecular weight (MM) ≤ 500 g/mol, (d) octanol-water partition coefficient (milog *P*) ≤ 5, and (e) topological polar surface area (TPSA) ≤ 140 Å^2^. [35]. The thiosemicarbazone TSC01 complied with Lipinski's rule, showing the probability of good bioavailability after oral administration (Table 1). Oral bioavailability represents a major obstacle in the development of drugs and various factors, such as aqueous solubility, dissolution rate, drug permeability, and first-pass metabolism, affecting the oral bioavailability [36].

Furthermore, the ability to predict, quickly and reliably, attributes of drug candidates, such as absorption, distribution, metabolism, excretion, and toxicity (ADMET), helps to exclude molecules with potential issues and assist researchers with syntheses and investigations of new compounds [37]. TSC01 presents theoretical blood-brain barrier and intestinal absorption, which suggests good absorption, probable intestinal permeability, and good solubility. Hypothetically, this compound does not act as a substrate and can inhibit some CYP450 isoforms, increasing the chances of drug interactions. Also, TSC01 has high inhibitory promiscuity and is not liable to active transport. Cytochrome P450 is an enzyme that catalyzes the metabolism of a wide variety of compounds, including xenobiotics and drugs [2, 33]. High interaction between drugs and cytochrome enzymes decreases drug efficiency [2, 33]. Furthermore, cytochrome P450 inhibition isoforms can cause drug interactions where coadministered drugs are not metabolized and toxic levels accumulate, known as drug-drug interaction [2, 33]. However, TSC01 seems not to inhibit CYP2D6, which promotes the metabolism of many drugs [2]. P450 enzymes generate reactive oxygen species (ROS) whose imbalance is responsible for interacting with proteins, DNA, and lipids leading to cell damage and death [14, 38]. TSC01 does not show theoretical genotoxic and carcinogenic capacity and exhibits a theoretical LD50 of 2.8508 mol/kg for rat acute toxicity that is considered as category 3 (DL50 > 500–5000), which is applicable for new drugs [39]. Rezki et al. (2018) had shown compounds with theoretical LD50 in rat acute toxicity ranging between 2.59 and 2.78 mol/kg to be nontoxic and safe. However, there is theoretical toxicity against fish, bees, and *Tetrahymena pyriformis* (Table 2). The effect of pollutants and chemicals on the ecosystem is a matter of great concern [40]. Advanced information regarding the environmental toxicity potential helps to prevent environmental disasters, because of high use of chemicals commercially, misuse, and improper disposal of chemical compounds [41].

### 3.2. *In Vitro*: Cytotoxicity

Cytotoxicity against human cell line is well known as an important predictive model for toxicity in humans [42]. Hep-G2 cells have low gene expression levels of cytochrome CYP450 enzymes and xenobiotic receptors; however, they are well-known hepatic cell lines that are easy to handle and cost-effective [43, 44]. In addition, HT-29 cells are commonly used as a human intestinal function model *in vitro* to study cell toxicity [45, 46]. The imbalance between ROS generation and the antioxidant activity of the cells promotes the oxidative stress responsible for cell damage and apoptosis [47]. Treatment with TSC01 did not reduce cell viability in Hep-G2 (tumor) or HT-29 (tumor) cell lines *in vitro* (Table 3). Many studies have proven that thiosemicarbazones have low or no cytotoxicity in normal cells, which indicates high selectivity rates [48]. Also, if we consider the biological activities already proven in the literature against *T. cruzi* (0.31 *μ*mol L^−1^) [21] and *C. albicans* and *C. krusei* and *C. glabrata* (13.12 *μ*mol L^−1^) [7], the selectivity index will be 322 and 7.6, respectively. The selectivity index measures the window between cytotoxicity and pharmacological activity. It is an indicator of safety and efficacy of compounds with pharmacological potential. The higher this index, the greater the potential use of these compounds in future clinical tests, with an SI above 3 being promising [49].

### 3.3. In Vivo Acute Toxicity Parameters

During drug development, it is vital to identify the toxic potential of chemical compounds [50, 51]. Exposure to chemicals can be harmful to humans, resulting in side effects [50]. The *in silico* and *in vitro* studies were used as a preliminary indicator of low toxicity. International guidelines support alternative procedures to replace and reduce animal testing [28, 51]. Acute nonclinical toxicity of the TSC01 substance was performed, which usually provides safety information, dose range, and possible side effects [52]. Toxic compounds promote the inflammation process, which is related to oxidative stress leading to cell damage [15]. At a dose of 300 mg/kg, stimulant signs of the central nervous system (CNS) were evidenced, such as increased hyperactivity and piloerection, and an increase in other behaviors such as walking, cleaning, climbing, lifting, shaking the head, and abduction of the hind. The admetSAR software predicted the ability of TSC01 to permeate the blood-brain barrier (Table 2). Also, signs related to the autonomic nervous system (ANS) such as defecation, diarrhea, and urination were increased. The feces showed a greenish color. All changes disappeared after 24 h. After the dose of 2000 mg/kg, similar behavior was observed in the animals that were treated with the dose of 300 mg/kg up to 1 h after treatment; depressive signs of the central nervous system were evidenced, with no greater intensity of effects, so we cannot correlate dose and acute toxic effects. There was also evidence of a decrease in behaviors, such as walking, cleaning, climbing, standing up, and shaking the head. However, abduction of the hind legs was still present. And constipation and increased urination were observed. Again, all changes disappeared after 24 hours of administration, which can be associated to a half-life of TSC01 due to the metabolic and excretion activity [52]. Behavioral screening helps during the evaluation of toxic potential [53]. ROS accumulation causes oxidative stress that induces behavioral changes in the CNS [54, 55]. Neuroinflammation caused by oxidative stress promotes signs of depression and anxiety [56]. During the 14 days of the experiment, no deaths were observed in any animal treated with a single dose of 300 mg/kg or 2000 mg/kg. Therefore, based on Annex I of Guide n. 423 of the OECD [28], it was possible to estimate the LD50 at 5000 mg/kg and the GSH 5 (low toxic or atoxic). Food intake, water consumption, and weight changes are essential parameters for safety studies that show the physiological status of the animal and work as an indicator of adverse metabolic effects [52, 57]. In relation to water consumption, there was a statistically significant increase in the animals treated with the dose of 2000 mg/kg (Table 4). In addition to this, the animals treated with a dose of 2000 mg/kg did gain weight in the first week; however, weight gain was less than the control.

**Figure 2:**
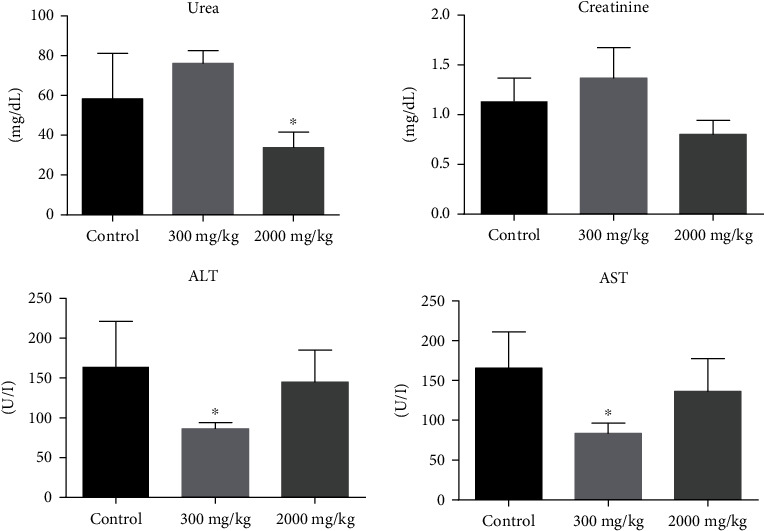
Effects of the acute treatment of TSC01 on the biochemical parameters of treated female Balb/c mice. The results are expressed as mean ± SD analyzed by ANOVA followed by Dunnett's test, ^∗^*P* < 0.05 (*n* = 6).

Toxic compounds cause damage and metabolic reactions to organs such liver, kidney, heart, spleen, and lung [58]. Some enzymes (GGT, ALP, AST, and ALT) are hepatic indicators for initial hepatic injury [59]. Urea and creatinine are nitrogenous metabolic substances that are increased when there is kidney damage [60]. The kidney and liver are predisposed to suffer oxidative damage and consequently cell damage [60, 61]. TSC01 decreased statistically the levels of ALT and AST in animals treated at doses of 300 mg/kg. Also, in animals treated at the dose of 2000 mg/kg, there was a statistically significant decrease in urea compared to the control (Figure 2). None of these changes are characteristic of toxicity, which was confirmed by histological analysis that did not demonstrate anatomical changes.

**Figure 3:**
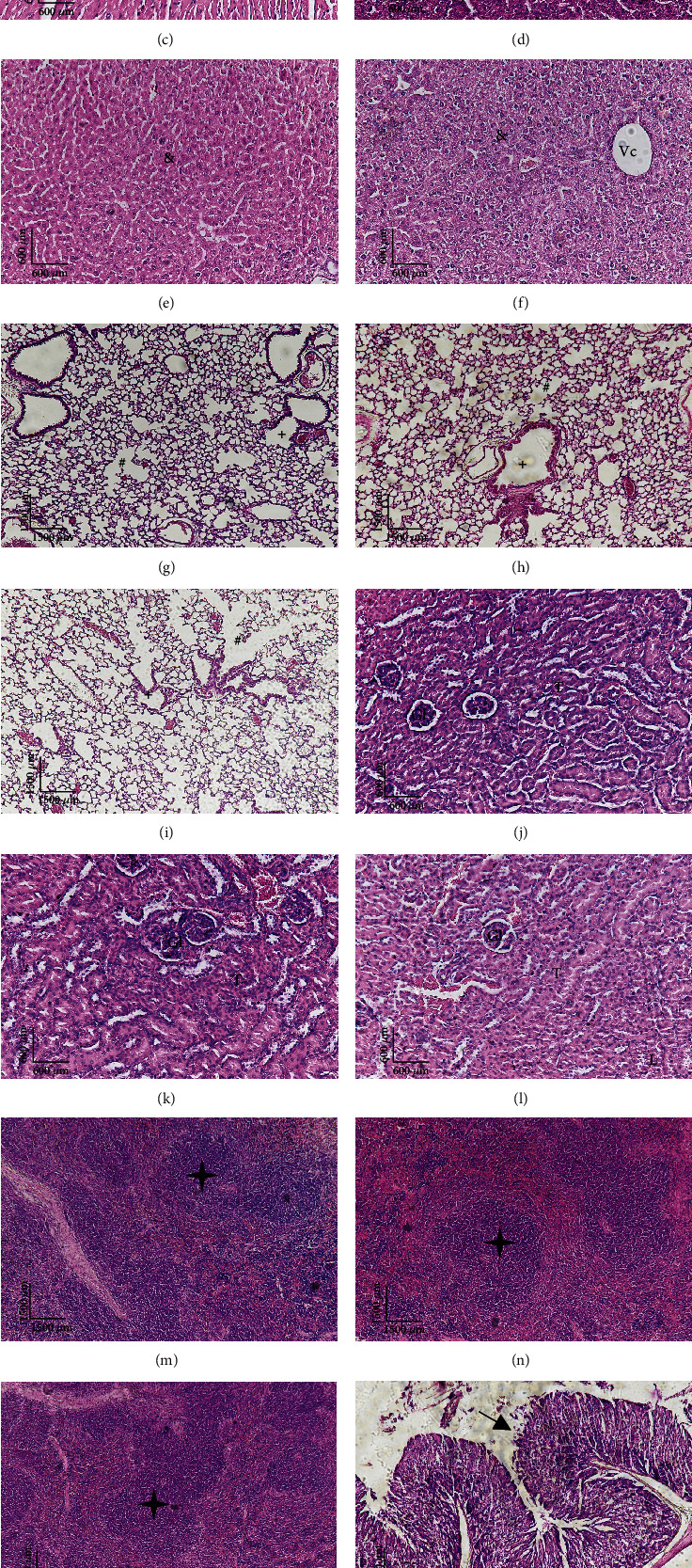
Histological sections stained in hematoxylin and eosin under different experimental conditions. (a, d, g, j, m, p) Control group—vehicle. (b, e, h, k, n, q) Group treated with a dose of 300 mg/kg. (c, f, i, l, o, r) Group treated with a dose of 2000 mg/kg. ∗: cardiomyocytes; Vc: central vein of the hepatic lobe; &: liver parenchyma; #: pulmonary alveoli; +: aerial and vascular branches; Gl: glomeruli; T: renal tubules; ✦: multiple primary lymphoid follicles, without an active germinal center; arrows: gastric pits.

The heart, stomach, liver, lung, spleen, and kidneys of animals submitted to doses of 300 and 2000 mg/kg of TSC01 were evaluated macroscopically and through histopathological examination. The organs did not present anatomical alterations, with statistically significant variations in the heart and liver index of the animals treated at the dose of 300 mg/kg (Table 5). A sign of organ damage is changes in organ weight [50]. However, no macroscopic alterations were observed in the organs of animals treated compared to the control, and the histological sections of the organs presented no notable tissue alterations (Figure 3). The heart exhibited unidirectional and uniformly distributed muscle bundles throughout the organ, showing cardiomyocytes of the same size and appearance (∗) in all described experimental conditions. In the liver, there were hepatocytes organized in cords present, going towards the central vein of the hepatic lobe (Vc). Hepatic triad and histological portal spaces were preserved. The parenchyma presented with cells of homogeneous size, shape, and organization (&). The lung presented clean and acellular pulmonary alveoli (#) and aerial and vascular (+) branches with standard histological appearance. In the kidney, glomeruli (Gl) were observed with preservation of the mesangial and capsule. Renal tubule (T) showed preservation of cells and tubular spaces. Vessels and juxtaglomerular apparatus all were preserved with standard histological appearance under all experimental conditions. In the spleen, there was the presence of multiple primary lymphoid follicles, without an active germinal center (✦). In the stomach, the presence of preserved gastric pits (arrow), vessels, and other components of the connective tissue, all of which have a standard histological appearance under all experimental conditions, was observed.

**Figure 4:**
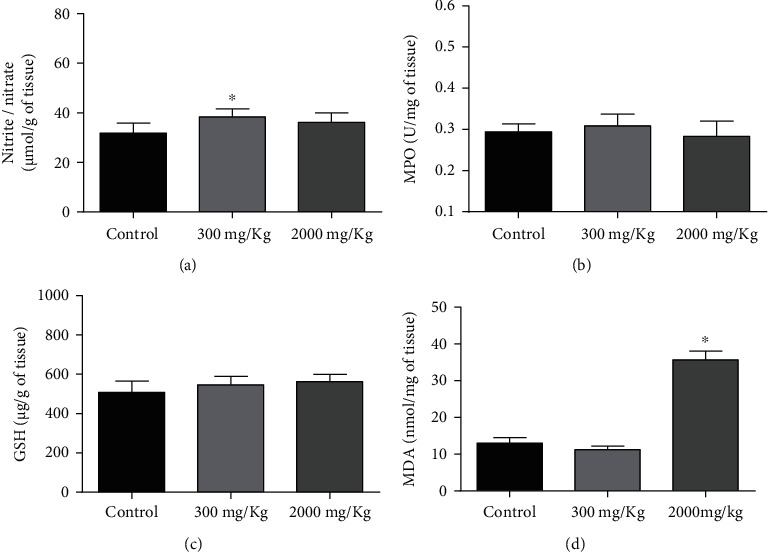
Determination of oxidative stress in the liver homogenate of treated female Balb/c mice: (a) dosage of nitrite, (b) dosage of myeloperoxidase (MPO), (c) dosage of glutathione (GSH), and (d) concentration of malondialdehyde (MDA). The results are expressed as mean ± SD analyzed by ANOVA followed by Dunnett's test, ^∗^*P* < 0.05.

Finally, the determination of oxidative stress in the liver was performed because the liver is the principal target of drug toxicity and generates reactive oxygen species (ROS) [62]. MDA is a final product of membrane lipid peroxidation and therefore is a biomarker [63]. The lipid peroxidation is a result of free radical-mediated injury [64]. Also, nitrite is the stable metabolite of nitric oxide (NO), having a direct correlation. NO is a biomarker of the inflammation process which leads to inhibition of hepatic proteins and the production of DNA that promotes cell injury [15, 61]. The TSC01 showed a significant increase in the production of MDA in the liver of animals treated with a dose of 2000 mg/kg and consequently an increase in lipid peroxidation and oxidative stress. Also, an increase in the production of nitrite in the liver of animals treated with the dose of 300 mg/kg was found (Figure 4). One of the most frequent side effects during the drug discovery is liver injury which can lead to withdrawal of new drug candidates [62].

## 4. Conclusion

In conclusion, our results suggest theoretical pharmacokinetic properties of promising oral bioavailability and low toxicity *in vitro* and *in vivo* for the studied compound. *In vivo*, TSC01 presented low toxic characteristics in acute nonclinical toxicity. In the liver, TSC01 produces lipid peroxidation and nitric oxide, which may involve toxic effects in this organ due to the development of oxidative stress. However, no anatomical changes were found in any organ, as their architectures were preserved without signs of inflammatory process. This study represents important knowledge about the toxicity of this compound and suggests future safe use. Due to the great pharmaceutical potential of this compound, more research is needed. Furthermore, this work followed international guidelines that are required for health agencies to prove safe use of drugs around the world.

## Figures and Tables

**Table 1:** Predicted *in silico*^∗^ parameters for the thiosemicarbazone TSC01.

Compound	milog *P*	MW	nALH	nDLH	nviolations	TPSA	nrotb
TSC01	2.69	304.81	4	2	0	49.31	5

^∗^Using Molinspiration software; milog *P*: octanol/water partition coefficient; nALH: number of hydrogen bond acceptor groups; nDLH: number of hydrogen bond donor groups; nviolations: number of violations; TPSA: topological polar surface area; nrotb: number of rotatable bands.

**Table 2:** ADMET properties for TSC01 calculated by the admetSAR software.

Model	Result	Probability
Absorption		
Blood-brain barrier	BBB+	0.7444
Human intestinal absorption	HIA+	0.8710
Caco-2 permeability	Caco2+	0.6079
P-glycoprotein substrate	Nonsubstrate	0.7056
P-glycoprotein inhibitor	Noninhibitor	0.6840
	Noninhibitor	0.9545
Renal organic cation transporter	Noninhibitor	0.6903
Distribution		
Subcellular localization	Mitochondria	0.7431
Metabolism		
CYP450 2C9 substrate	Nonsubstrate	0.7205
CYP450 2D6 substrate	Nonsubstrate	0.8450
CYP450 3A4 substrate	Nonsubstrate	0.6216
CYP450 1A2 inhibitor	Inhibitor	0.8960
CYP450 2C9 inhibitor	Noninhibitor	0.5897
CYP450 2D6 inhibitor	Noninhibitor	0.7296
CYP450 2C19 inhibitor	Inhibitor	0.8249
CYP450 3A4 inhibitor	Inhibitor	0.6117
CYP inhibitory promiscuity	High CYP inhibitory promiscuity	0.8773
Excretion and toxicity		
Human ether-a-go-go-related gene inhibition	Weak inhibitor	0.8621
	Noninhibitor	0.8652
AMES toxicity	Non-AMES toxic	0.6435
Carcinogens	Noncarcinogens	0.7408
Fish toxicity	High FHMT	0.9966
*Tetrahymena pyriformis* toxicity	High TPT	0.9920
Honey bee toxicity	Low HBT	0.8145
Biodegradation	Not ready biodegradable	1.0000
Acute oral toxicity	III	0.5041
Carcinogenicity (three-class)	Nonrequired	0.5814
ADMET predicted profile—regression		
*Model*	*Value*	*Unit*
Absorption		
Aqueous solubility	-3.7207	logS
Caco-2 permeability	1.2948	logPapp, cm/s
Toxicity		
Rat acute toxicity	2.8508	LD50, mol/kg
Fish toxicity	1.2528	pLC50, mg/L
*Tetrahymena pyriformis* toxicity	1.4066	pIGC50, *μ*g/L

**Table 3:** Cytotoxicity (CC_50_) of TSC01 against human cell line.

Compound	Hep-G2	HT-29
	CC_50_ (*μ*mol L^−1^)	CC_50_ (*μ*mol L^−1^)
TSC01	>100^∗^	>100^∗^

Cytotoxicity of thiosemicarbazone was evaluated against Hep-G2 and HT-29 cells after 24 h treatment. The CC_50_ values were calculated using Prisma Software. ^∗^At maximum soluble concentration (100 *μ*M), values in reduction of cell viability did not reach more than 50% and the CC_50_ values could not be determined. The data shows three independent experiments in triplicate.

**Table 4:** Effects of acute treatment of TSC01 on water consumption, feed intake, and weight change of treated female Balb/c mice.

Groups	Dose (mg/kg)	Water (mL)	Food (g)	Weight gain (g) (day 7)	Weight gain (g) (day 14)
Control	—	49.71 ± 5.37	24.57 ± 1.60	1.600 ± 0.55	0.5 ± 0.55
Treated	300	49.71 ± 6.46	23.29 ± 3.31	1.333 ± 0.52	0.5 ± 0.55
Treated	2000	63.29 ± 7.08^∗^	25.21 ± 4.17	0.6667 ± 0.52^∗^	1.75 ± 0.5^∗^

The results are expressed as mean ± SD analyzed by ANOVA followed by Dunnett's test, ^∗^*P* < 0.05 (*n* = 6).

**Table 5:** Effects of the acute treatment of TSC01 on the organ index of treated female mice.

	Control	300 mg/kg	2000 mg/kg
Heart index (mg/g)	4.52 ± 0.25	4.96 ± 0.34^∗^	4.90 ± 0.22
Lung index (mg/g)	15.96 ± 3.93	19.22 ± 2.81	20.78 ± 3.64
Stomach index (mg/g)	8.90 ± 1.32	8.83 ± 1.48	9.46 ± 1.52
Liver index (mg/g)	48.20 ± 4.14	41.36 ± 3.83^∗^	50.55 ± 3.84
Spleen index (mg/g)	5.86 ± 0.79	5.62 ± 0.59	6.09 ± 0.65
Kidney index (mg/g)	13.6 ± 0.38	13.8 ± 1.1	13.7 ± 1.29

The results are expressed as mean ± SD analyzed by ANOVA followed by Dunnett's test, ^∗^*P* < 0.05 (*n* = 6).

## Data Availability

No data were used to support this study.
